# Post-Translational Modifications of Nitrate Reductases Autoregulates Nitric Oxide Biosynthesis in Arabidopsis

**DOI:** 10.3390/ijms22020549

**Published:** 2021-01-07

**Authors:** Álvaro Costa-Broseta, MariCruz Castillo, José León

**Affiliations:** Instituto de Biología Molecular y Celular de Plantas, Consejo Superior de Investigaciones Científicas Universidad Politécnica de Valencia, 46022 Valencia, Spain; tyuhan@gmail.com (Á.C.-B.); macaslo3@ibmcp.upv.es (M.C.)

**Keywords:** cysteine S-nitrosation, nitrate assimilation, nitric oxide, nitrite reductase, tyrosine nitration, plant growth, ubiquitylation

## Abstract

Nitric oxide (NO) is a regulator of growth, development, and stress responses in living organisms. Plant nitrate reductases (NR) catalyze the reduction of nitrate to nitrite or, alternatively, to NO. In plants, NO action and its targets remain incompletely understood, and the way NO regulates its own homeostasis remains to be elucidated. A significant transcriptome overlapping between NO-deficient mutant and NO-treated wild type plants suggests that NO could negatively regulate its biosynthesis. A significant increase in NO content was detected in transgenic plants overexpressing NR1 and NR2 proteins. In turn, NR protein and activity as well as NO content, decreased in wild-type plants exposed to a pulse of NO gas. Tag-aided immunopurification procedures followed by tandem mass spectrometry allowed identifying NO-triggered post-translational modifications (PTMs) and ubiquitylation sites in NRs. Nitration of tyrosine residues and S-nitrosation of cysteine residues affected key amino acids involved in binding the essential FAD and molybdenum cofactors. NO-related PTMs were accompanied by ubiquitylation of lysine residues flanking the nitration and S-nitrosation sites. NO-induced PTMs of NRs potentially inhibit their activities and promote their proteasome-mediated degradation. This auto-regulatory feedback loop may control nitrate assimilation to ammonium and nitrite-derived production of NO under complex environmental conditions.

## 1. Introduction

Plants produce nitric oxide (NO) mainly through reductive pathways from nitrite [1]. An oxidative pathway from arginine has been proposed to be also functional in plants. However, NO synthase (NOS)-like proteins with the features of the animal enzymes have only been identified in some green algae [2,3]. Therefore, the functionality of such a kind of NO biosynthetic pathway remains controversial in plants [4]. The reductive pathway from nitrite requires the previous reduction of nitrate catalyzed by NAD(P)H- and molybdenum (Mo)-dependent nitrate reductases (NRs) [5]. NRs, as well as other molibdoproteins, can also reduce nitrite to NO [6,7]. Nitrite can also be reduced to NO by the action of the mitochondrial electron transport machinery under hypoxia [8]. Because of its elevated toxicity, plants rapidly reduce nitrite to ammonium by soluble stroma-localized chloroplastic ferredoxin:nitrite reductases (NiRs). The balance between NR and NiR activities seems to be crucial to determine the amount of NO that plants can produce and accumulate. Arabidopsis thaliana has two NRs called NR1/NIA1 and NR2/NIA2 [9,10] and a single NiR1, which upon CRISPR/Cas9-mediated knocking-out leads to enhanced NO accumulation [11]. NR function is regulated by post-translational modifications [12,13], being the reversible phosphorylation and proteolysis the better documented [14]. Both phosphorylation and proteolysis act as negative regulators of NRs [15,16]. Arabidopsis NIA1 and NIA2 are also modified by sumoylation involving the E3 SUMO ligase activity of AtSIZ1 that increases their activities [17].

NRs are enzymes with multiple redox centers that are required for the proper electron transfer from the physiological electron donors to the oxidized substrates [18]. NRs are homodimers composed of 100 kDa polypeptide subunits and three redox cofactors FAD, iron-heme, and molybdopterin (MoCo) [18,19]. Several domains have been defined for NR. Two central cleavable regions separate the N-terminal molybdopterin binding domain close to the dimerization interface region, the cytochrome b heme domain in between cleavage sequences, and the C-terminal Flavin/NADH binding domain [18]. Three C residues seem to be critical for binding MoCo [19] and for dithiol-based dimerization [20]. A fourth C conserved residue in plant NRs is located at the NADH-binding domain and seems to be important for the cofactor binding.

## 2. Results

### 2.1. Exogenously Applied NO Represses the Endogenous Biosynthesis Acting on NRs

**Figure 1:**
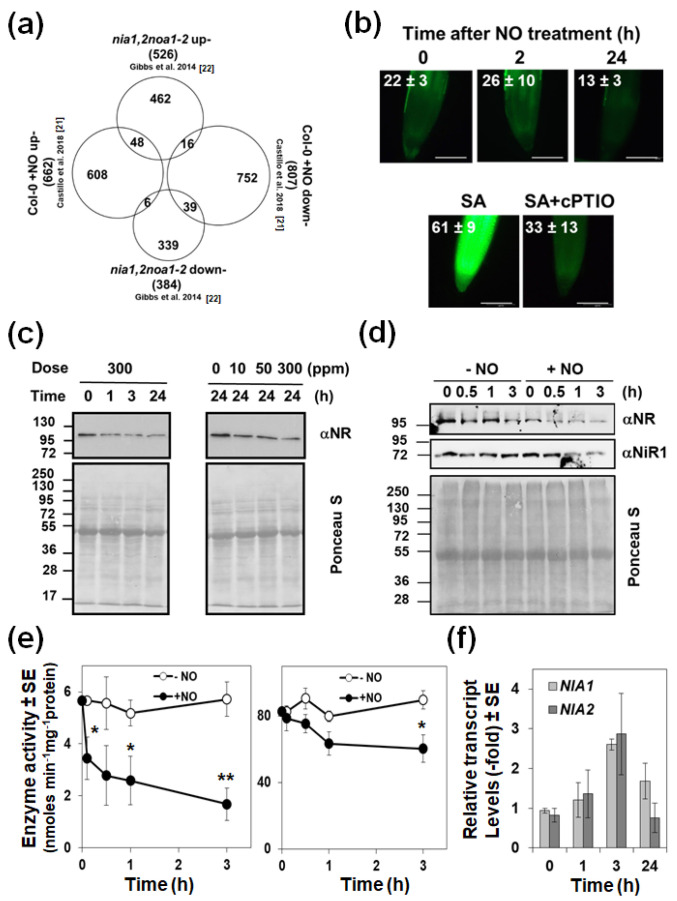
Exogenous NO negatively mimics endogenous NO deficiency. (**a**) Venn diagram showing the overlaps between up and down-regulated genes in NO-deficient *nia1,2noa1-2* mutant plants and wild-type plants exposed to 300 ppm of NO gas. (**b**) Endogenous NO levels in wild-type roots were analyzed by treatment with DAF-FM DA fluorophore and images are representative of each condition. Positive and negative controls with salicylic acid (SA)-treated and NO scavenger cPTIO-treated roots, respectively, are shown. Values are the mean ± standard error of three independent replicates. Scale bars correspond to 100 μm. (**c**) Levels of NR protein in plants exposed to increasing NO concentrations and different times were detected by Western blot with an anti-NR antibody. (**d**) NR and NiR1 protein levels at early times after plants exposure to NO were detected by Western blot with an anti-NR and anti-NiR1 antibodies. Loading control is shown by Ponceau S stained membranes. The position of protein size markers (kDa) is shown on the left side. (**e**) Levels of NR and NiR activities in NO-exposed (+NO) and mock control (−NO) plants were quantified from two technical replicates for each of four independent biological replicates. (**f**) Transcript levels were measured by qRT-PCR with specific primers for *NIA1* and *NIA2* genes and *ACT2* as a housekeeping gene and made relative to the levels detected at 0 time. Values are the mean of three independent replicates ± standard error. * *p* < 0.05 and ** *p* < 0.005 in Student’s t-test comparing +NO versus −NO at each time.

**Figure 2:**
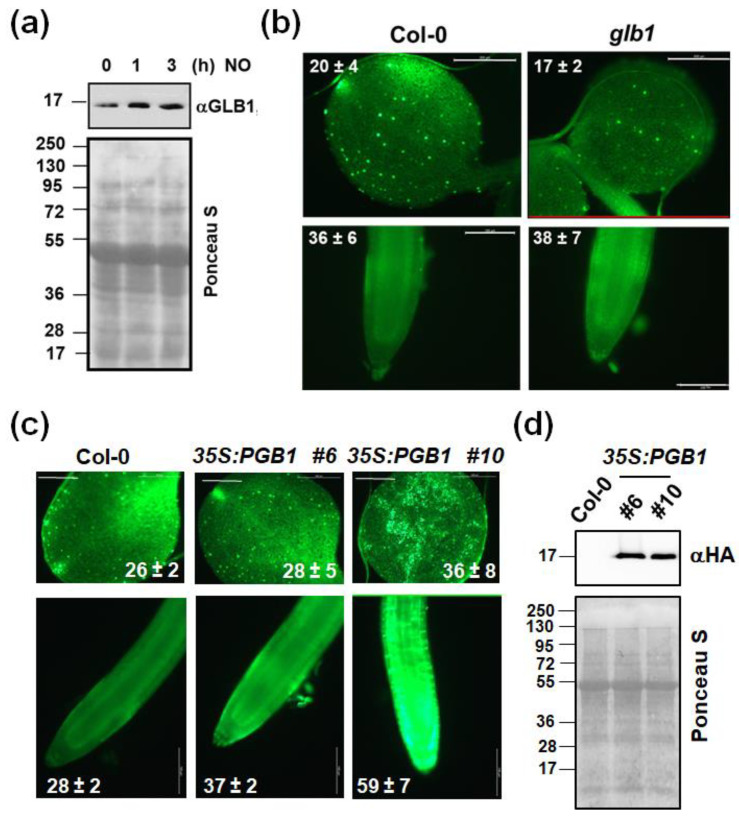
Effect of NO on GLB1/PGB1 protein levels and endogenous levels of NO in wild type, *glb1* mutant, and *PGB1* over-expressing transgenic plants. (**a**) GLB1/PGB1 protein levels at early times in hours (h) after plant exposure to NO were detected by Western blot with an anti (α)-GLB1,2 antibody (Agrisera). Loading control is shown by Ponceau S stained membrane. The position of protein size markers (kDa) is shown on the left side. (**b**,**c**), Cotyledon (upper panels) and roots (lower panels) of the indicated mutant and transgenic overexpressing plants after treatment with DAF-FM diacetate. NO content values inside each panel are the average of 3 to 6 independent samples ± SE. White bars represent 500 and 100 μm for cotyledon and root images, respectively. (**d**) GLB1/PGB1 protein levels in HA-tagged *PGB1* over-expressing transgenic plants were detected by Western blot with anti (α)-HA-HRP antibody (Thermo Scientific). Loading control is shown by Ponceau S stained membrane. The position of protein size markers (kDa) is shown on the left side.

**Figure 3:**
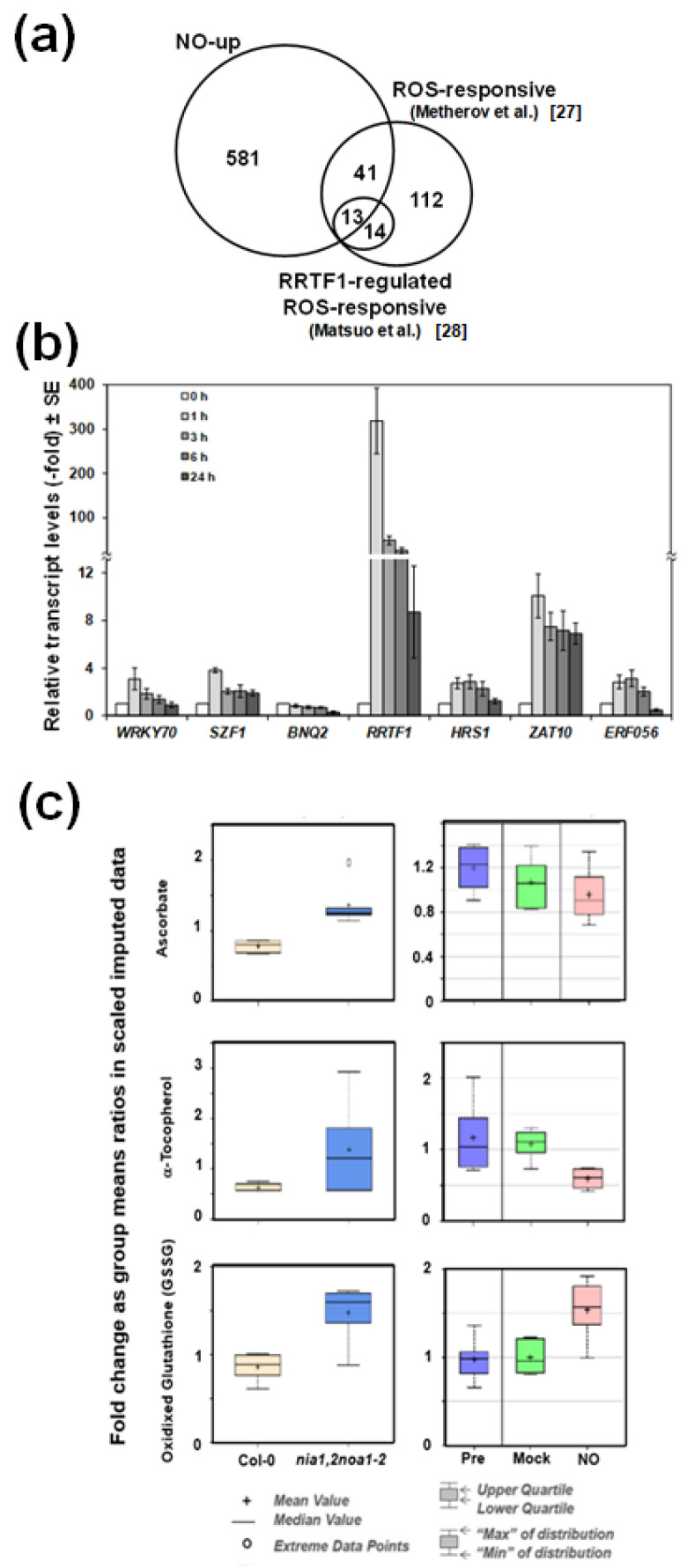
Effect of NO on the redox status of the plants. (**a**) Venn diagram showing the overlaps between NO up-regulated, ROS-responsive, and RRTF1-regulated genes. (**b**) Levels of ROS-related transcription factor encoding genes were analyzed by qRT-PCR with RNAs isolated, at the indicated times, from NO-exposed Col-0 plants. Values are the mean ± standard error of three independent biological replicates. (**c**) The levels of antioxidants were analyzed by LC-MS in Col-0 and the NO-deficient *nia1,2noa1-2* mutant plants (left boxes) and wild type Col-0 plants (right boxes) before (Pre) and after 6 h exposure to 300 ppm NO (NO) or control untreated (Mock). Values are the mean of six independent biological replicates. The boxes represent the mean and median values with the upper and lower quartile limits as well as the maximum and minimum of the distribution and extreme data points.

Wild type plants treated with NO gas and untreated NO-deficient mutant plants would be expected to display oppositely regulated responses. However, when comparing the transcriptome of NO-treated plants [21] to that of NO-deficient mutant plants [22], we found 87 genes that were similarly regulated (Table S1) and only 22 oppositely regulated (Figure 1a). This paradox suggests that NO-treatment is somehow leading to endogenous NO-deficiency. Such an effect may be the result of repressing NO production or inducing its metabolism and/or scavenging. By exposing plants to a pulse of exogenous NO gas and subsequent treatment with DAF-FM DA, we found that the fluorescence associated with endogenous NO decreased at 24 h (Figure 1b). This response is preceded by a dose-dependent decrease in the NR protein (Figure 1c) that is detected as soon as 30 min after exposure to NO, and only slightly affect on NiR1 protein levels at longer times (Figure 1d). Concomitantly, the NADH-NR activity dropped down, starting already at 5 min in NO-exposed plants, whereas NiR activity did not change at this time (Figure 1e). After 3 h after exposure to NO, more than 70% of the NR activity and less than 30% of the NiR activity were lost (Figure 1e). By using other electron donors different than NADH, such as dithionite-reduced methyl viologen (MVH) or bromophenol blue (BPBH), the terminal NR activity was also assayed [23]. MVH-NR and BPBH-NR were found to be inactivated by NO though the inhibitory effect on BPBH-NR was only detected after 3 h after NO treatment (Supplementary Materials Figure S1). By measuring the transcript levels of the two genes coding for NRs in arabidopsis, we confirmed that the reduced NR protein and activity levels triggered by NO could not be due to reduced *NIA1* and/or *NIA2* gene expression. In fact, we detected a transient upregulation of both genes by 3 h after exposure to NO and further downregulation back to the levels detected in untreated plants (Figure 1f). These data suggest that exogenous NO led to a reduction in NO content mainly by repressing the endogenous NO production through inhibition/degradation of NRs. However, these data do not rule out the possibility that the reduced NO content in plants exposed to exogenous NO may also be due to increased scavenging or metabolism. Regarding scavenging, phytoglobins such as GLB1, are very efficient NO scavengers that bind it to the iron of their heme group [24]. Table S1 shows that the GLB1/HB1 gene, renamed as Phytoglobin1 (PGB1) [25], was one of the genes up-regulated both in NO-treated Col-0 (6.5-fold) and *nia1,2noa1-2* (3-fold) plants above the levels in untreated wild type plants. Accordingly, we found that the levels of GLB1/PGB1 protein were increased upon exposure of plants to NO (Figure 2a). We have generated plants overexpressing *GLB1/PGB1* gene and, together with *glb1* mutants, were analyzed for their basal endogenous NO content. Neither *glb1* mutant plants contained higher content nor *35S:PGB1* transgenic plants showed significantly reduced NO levels compared to wild type plants (Figure 2b,c), as it would be expected if PGB1 was scavenging NO efficiently. On the contrary, we found a slight increase in NO content in some of the analyzed transgenic lines (Figure 2c), which may be due to the reported function for heme groups as catalytic centers for nitrite reduction under certain conditions [26]. These findings suggest that PGB1-mediated scavenging is not decisive in determining NO endogenous levels. Alternatively, NO could also be metabolized by reaction with oxidative species. We found that exposure of plants to a NO pulse triggers an oxidative response. The *MADR6* gene coding for monodehydroascorbate reductase 6 and the *CAT2* gene coding for catalase 2 were strongly up and down-regulated, +12.1-fold and −4.1-fold respectively, upon NO exposure [21]. The *RRTF1* gene coding for the Redox Responsive Transcription Factor 1 is the second most up-regulated (17-fold) gene in plants exposed to NO [21]. We found an overlapping between the NO-responsive and ozone-treated transcriptomes (Figure 3a). Besides, 54 out of the 180 previously reported ROS marker genes [27] and 13 out of the 27 ROS marker genes identified as up-regulated in plants over-expressing the RRTF1 gene [28] were up-regulated in NO-exposed plants (Figure 3a). We confirmed by RT-qPCR that the *RRTF1* gene, as well as other oxidative response-related transcription factor encoding genes such as ZAT10, SZF1, ERF056, HRS1, and WRKY70, were strongly up-regulated by 1 h after NO exposure (Figure 3b). Oxidative responses may result from the altered function of antioxidant systems. Ascorbate, glutathione, and α-tocopherol are the most relevant antioxidant systems in plants [29], so we analyzed the levels of those metabolites in NO-deficient mutant plants and NO-treated wild type plants. Ascorbate and α-tocopherol showed opposite patterns of accumulation in both.

Regarding conditions (Figure 3c), however, the levels of oxidized glutathione (GSSG) increased similarly in both NO-deficient and NO-treated plants (Figure 3c). These findings suggest that the relevance of antioxidant systems in modulating the endogenous NO levels cannot be ruled out.

### 2.2. The Endogenous NO Levels Are Mainly Controlled by Post-Translational Modifications of NRs

**Figure 4:**
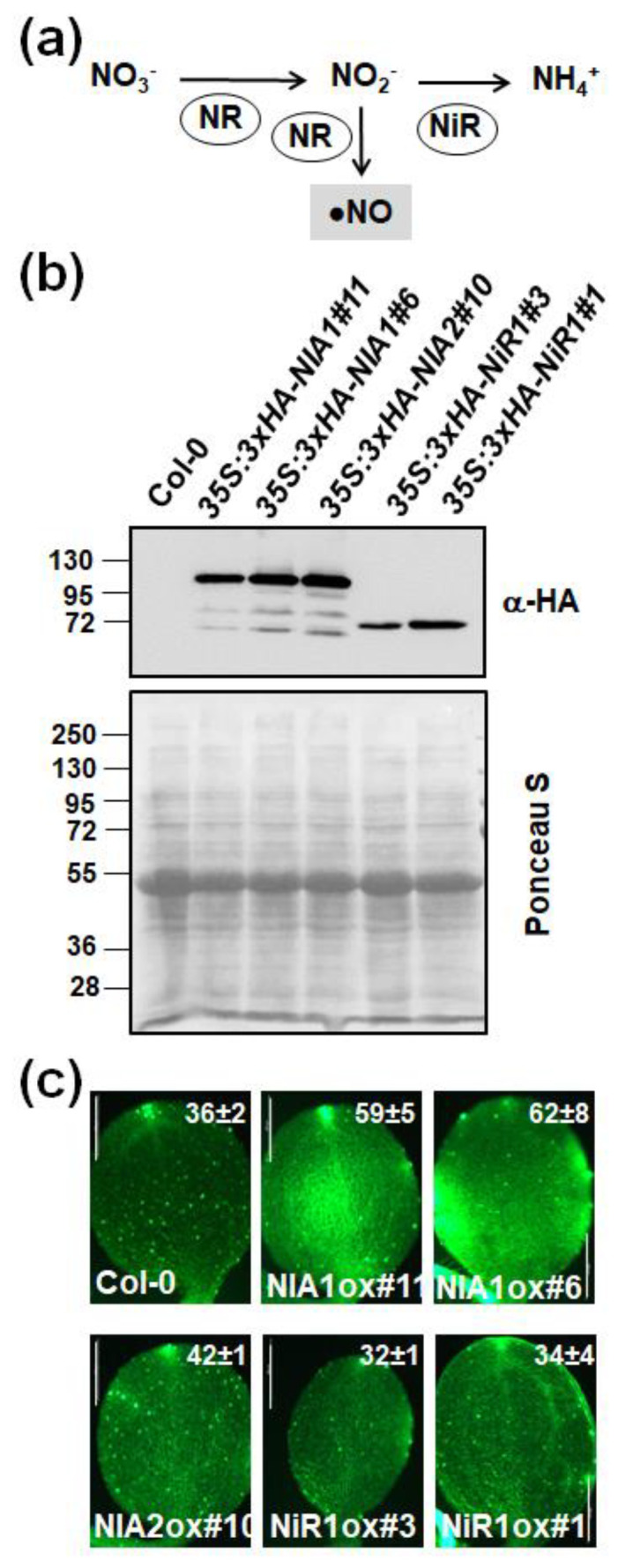
Levels of protein and endogenous NO content in plants overexpressing HA-tagged NIA1, NIA2, and NiR1 proteins. (**a**) Scheme showing nitrate assimilation pathway catalyzed by nitrate reductases (NR) and nitrite reductase (NiR) and the alternative reduction of nitrite to NO. (**b**) Levels of HA-tagged proteins were detected by Western blot with an anti-HA antibody coupled to horseradish peroxidase. Loading control is shown with Ponceau S stained membranes. The position of protein size markers (kDa) is shown at the left side. (**c**) Endogenous levels of NO in cotyledons of the indicated genotype after treatment with DAF-FM diacetate. Values are the mean ± standard error of three independent replicates. Scale bars correspond to 500 μm.

NO production in plants occurs mainly through nitrite reduction in a process that can be considered as a side branch of the nitrate assimilation pathway (Figure 4a). By generating; transgenic plants overexpressing any of the two arabidopsis NR encoding *NR1/NIA1* and *NR2/NIA2* genes, we have confirmed that NRs mediate NO biosynthesis. Only overexpression of *NR1/NIA1* but not *NR2/NIA2* led to a significant increase in NO levels compared to untransformed plants (Figure 4c), despite both expressed similar levels of transgenic protein (Figure 4b). We also generated lines overexpressing *NiR1* gene (Figure 4b) that showed no significant alterations in NO content (Figure 4c), thus suggesting NiR1 certainly did not catalyze the reduction of nitrite to NO.

**Figure 5:**
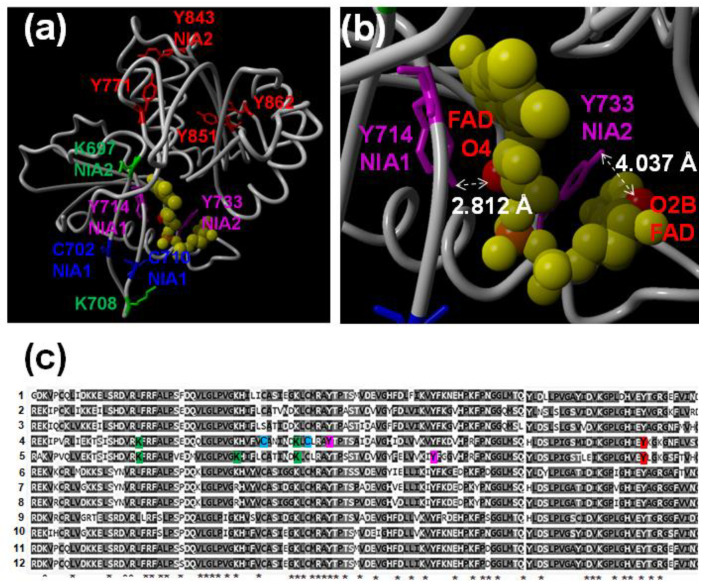
Location of the identified nitrated Y (red), aminated Y (magenta), S-nitrosated C (blue), and ubiquitylated K (green) residues of NIA1 and NIA2 proteins in the FAD-binding domain. (**a**) Post-translationally modified residues identified in arabidopsis were located based on homology sequence in the 3D structure model of the corn nitrate reductase (PDB code 2cnd) by using Yasara view application. FAD is colored in yellow. (**b**) Detail of the FAD-binding pocket showing the distance from the hydroxyl group of Y aromatic ring and the O2B and O4 atoms of the FAD molecule. (**c**) Degree of conservation of the identified modified amino acids in NRs from Rice (1, 6, 11 and 12 correspond to Os10g17780.1, Os02g53130.1, Os08g36480.1, Os08g36500.1), arabidopsis (4, and 5 corresponds to AtNIA1and AtNIA2), Populus tritocharpa (2,3 correspond to POPTR_0005s16180, and POPTR_0002s089300), and maize (7, 8, 9, 10 correspond to GRMZM2g428027, GRMZM5g878558, GRMZM2g076723, and GRMZM2g568636) plants. Sequence alignment was performed with Clustal omega. * below are fully conserved amino acids in NRs from different plants.

The transgenic lines over-expressing HA-tagged versions of NR1 and NR2 were used for immunopurification with anti-HA-coated magnetic beads followed by LC-MS/MS proteomic analyses (IP-MS). We identified multiple NR1 and NR2 PTMs, including ubiquitylation of K residues, S-nitrosation of C residues, and nitration or amination of Y residues (Tables S2 and S3). Some of the PTMS were detected in both NR1/NIA1 and NR2/NIA2, whereas others were identified only in one of them (Figure S2). In addition, we found PTMs in domains involved in binding prosthetic groups but also out of these domains (Figure S2). Inside the cytochrome b5 heme-binding domain, we identified nitrated Y548 and Y614 and S-nitrosated C573 of NR1/NIA1, and nitrated Y545 of NR2/NIA2 (Figure S2). In the FAD-binding domain, the C702 and C710 of NR1/NIA1 were identified as S-nitrosated; the Y714 and Y771 of NR1/NIA1 and the Y714 and Y771 of NR2/NIA2 were identified as aminated and nitrated, respectively (Figure S2). Taking advantage of the 3D structure of the FAD-binding domain of corn nitrate reductase (PDB code 2cnd) and based on the sequence similarity with arabidopsis NR/NIAs, we have located these PTMs in the 3D structure and found that both nitrated Y771 and S-nitrosated C702 and C710 are relatively far from the FAD binding site and not oriented towards the FAB binding site (Figure 5a). In turn, the aminated Y714 (NIA1) and Y733 (NIA2) were located at 2.8–4.0 Å and directed towards the FAD molecule (Figure 5b). We have also found the corresponding peptides with those residues nitrated although with a lower score, likely due to the instability of nitro groups easily reduced to amino groups. All these modified Y and C residues are fully conserved in NRs from other plants (Figure 5c). Among amino acids that were differentially modified in both arabidopsis NRs, we identified the S-nitrosation of NR2/NIA2 C191, which is the amino acid involved in molybdenum cofactor (MoCo) binding (Figure S2).

## 3. Discussion

The mechanism by which NO is synthesized in plants remains controversial and incompletely understood [1,30,31]. Although oxidative mechanisms might participate in NO production, it seems well established that most of the NO is synthesized through reductive mechanisms from nitrite [6]. Here, we report an approach based on the generation of arabidopsis NR overexpressing plants and their use in IP-MS to analyze how NO may regulate nitrate assimilation enzymes through PTMs. The overexpression of NR1/NIA1 and, to a lesser extent NR2/NIA2, led to increasing NO accumulation (Figure 4c), thus suggesting NR/NIAs are involved in NO production. It has been reported that PTMs, mainly reversible phosphorylation and sumoylation of a key K residue, have a relevant role in controlling NR activity and protein stability [13,17,32]. However, much less is known about other PTMs affecting NR function. Here we describe the *in planta* identification of K ubiquitylation, Y nitration or amination, and C S-nitrosation sites in NR1 and NR2 arabidopsis proteins (Figure S2). Some of these modifications affected Y residues (NIA1 Y714 and NIA2 Y733) very close to the FAD binding site (Figure 5b), thus likely interfering with the efficient binding of FAD by the enzyme, a process that is essential for the activity. Moreover, the S-nitrosation of NIA2 C191, which is involved in binding the essential molybdenum cofactor (Figure S2), can prevent the MoCo-apoenzyme assembly, thus avoiding the proper electron transference mechanisms essential for the redox activity of the enzyme. It is worth noting that S-nitrosation of C191 may represent a differential pattern of post-translational regulation for the constitutive NR2/NIA2 enzyme versus the inducible NR1/NIA1 [33]. A larger contribution of NR1/NIA1 relative to NR2/NIA2 to NO synthesis in arabidopsis has also been recently reported [34]. Other nitrated and/or aminated Y residues were identified in both NR/NIAs (Tables S2 and S3 and Figure S2). A cluster of two Y located close to K356/K353 residues (in NR1/NR2, respectively), which is sumoylated [17], was found nitrated only in NR1/NIA1 (Figure S2), thus pointing to nitration likely interfering with its sumoylation-mediated activation. Unfortunately, the lack of models for the 3D structure of the whole NR enzyme does not allow predicting the effect of these modifications on the activity. The only 3D structure model available for the FAD-binding domain of the corn NR allowed us to locate, by homology, the position of NIA1 Y714 and NIA2 Y733 in the structure (Figure 5b). Both residues are very close to the FAD pocket. The addition of a nitro or amino group to the aromatic ring of Y will perturb the volume and charge of the amino acids and will likely hamper the access of the cofactor with the consequent negative effect on activity. It is worth mentioning that NO can inactivate NR activity using NADH, MVH, and BPBH as electron donors (Figure S1), thus suggesting nitration/amination of Y close to FAD as well as S-nitrosation of C involved in binding MoCo are both relevant in the NO-trigger inactivation of NR proteins. In any case, the negative or positive effect of nitration, amination, or S-nitrosation of these residues on NR activity would require further experiments with in vitro nitration of recombinant NR proteins, and the subsequent analysis of NR activity to be confirmed. Our attempts to perform this experimental approach have been unsuccessful as the expression of full-length active recombinant NRs is a very challenging task that has, to our knowledge, not been reported yet.

In the eukaryotic algae *Chlamydomonas reinhardtii*, it has been solidly supported that NR-dependent NO synthesis occurs by supplying electrons from NAD(P)H to NR FAD-Heme cofactors, and from them to the molybdoenzyme NO-forming nitrite reductase (NOFNiR), which is homologue to the human amidoxime reducing component (ARC) [35]. Regarding this, NO-triggered post-translational modifications of Y residues close to the FAD cofactor (Figure 5a and Figure S2) could be relevant to modify the NR interaction with ARC proteins with NOFNiR activity. However, we have evidence suggesting that a scenario like that is not likely operating in arabidopsis. We checked that the endogenous NO levels were not altered in arabidopsis mutant plants carrying T-DNA insertions in the two arabidopsis genes homologue to those coding for ARC/NOFNiRs in Chlamydomonas (data not shown). Besides, the NO synthesis mediated by NR:NOFNiR would be essentially independent of the NR Moco function [35], and our data suggests that NO might inactivate NRs through post-translational modifications in amino acids located both at the FAD and Moco domains (Figure 5a and Figure S2), thus exerting its negative regulation on both global and terminal NR activities (Figure S1).

NRs were found to be also ubiquitylated in several K residues (Tables S2 and S3 and Figure 5). Protein ubiquitylation can modify several features of the target protein depending on the site and degree of ubiquitylation. Polyubiquitylated proteins are usually targeted for proteasome-mediated degradation [36]. We found that the levels of NR protein decreased after plant exposure to exogenous NO gas (Figure 1c,d). Treatment of plants with the proteasome inhibitor MG132 led to NR protein accumulation over the levels detected in untreated control plants, and the NO-triggered NR degradation was also partially rescued by MG132 (Figure S3a). These effects are specifically exerted on NR proteins as no significant changes in neither *NIA1* nor *NIA2* gene transcripts were detected in plants treated with MG132 or NO (Figure S3b). It is thus likely that the ubiquitylation sites detected in NRs are associated with polyubiquitylation and degradation, thus leading to lower NO production. We already reported the coexistence of ubiquitylation with nitration and S-nitrosation as a mechanism to attenuate ABA signaling through nitration-mediated inactivation and further proteasome-mediated degradation of ABA receptors [37]. A similar mechanism based on post-translational modification and proteasome-mediated degradation of NO-biosynthetic enzymes would function as a fine-tuning negative autoregulatory loop controlling NO homeostasis.

## 4. Materials and Methods

### 4.1. Plant Material

Arabidopsis seeds were surface sterilized with chlorine gas before sowing in MS-MES media plates containing 1% sucrose. Plants over-expressing HA-tagged versions of NR1, NR2, NiR1, and PGB1 were generated by subcloning the full-length cDNAs in pAlligator 2 vector, and further transformation of *Agrobacterium tumefaciens* C58 with the corresponding constructs. Plants were then genetically transformed by dipping floral organs in a suspension of transformed Agrobacterium [38] and selected for homozygotic transgenes by screening fluorescence in seeds.

### 4.2. RNA iIsolation and RT-qPCR

Plants grown in vitro under long days (16 h light/8 h darkness) conditions for 10–14 days were exposed to a pulse of NO (300 ppm, 5 min). At the indicated times, total RNA was extracted and purified with Nucleospin RNA Plant kit (Macherey-Nagel, Dueren, Germany), reverse transcribed with M-MuLV Reverse transcriptase (RNase H minus) and oligodT, and the resulting cDNAs quantified by real-time PCR with ABI 7500 Fast Real-Time Thermocyclers by using specific primer pairs (Table S4). Three biological replicates of RNA with three technical replicates for each were processed by qRT-PCR, and the values are the mean ± standard error.

### 4.3. Western Blot Analyses

The levels of NR and NiR1 protein were analyzed in total protein extracts by SDS-PAGE, semidry blotting onto nitrocellulose membranes and further probing with polyclonal anti-NR (1:1000 dilution; Agrisera, Vännäs, Sweden), anti-NiR1 (1:1000 dilution) antibodies [39], and anti-HA-HRP (1:1000 dilution; Invitrogen, Carlsbad, CA, USA). Whole 10–14 day seedlings grown as described above were used for protein extraction. Quantification of protein was performed by using Bradford reagent (Bio-Rad, Madrid, Spain). The loading control was assessed by staining nitrocellulose membranes after blotting with Ponceau S. The whole stained membranes are shown to ensure equal loading. Commonly used housekeeping proteins, such as tubulin or actin, could not be used as loading controls as they were responsive to NO-related processes on microtubule organization as recently reviewed [40]. The Western blots shown are representative of two to four independent replicates.

### 4.4. Proteomic Analyses of HA-Tagged Overexpressing Plants

Total crude protein extracts were prepared by grinding liquid nitrogen-frozen samples and further extraction in 50 mM Tris-HCl buffer, pH 8.0, containing 150 mM NaCl, 5% glycerol, 5 mM EDTA and 0.05% (*v*/*v*) Triton X-100. Immunopurification with antiHA-magnetic beads (Miltenyi Biotec, Gladbach, Germany) and elution under non-denaturing nonreducing conditions as well as LC-MS/MS-based proteomic analyses were performed as previously reported [37] from proteins extracts obtained from plants expressing HA-tagged versions of NRs. Details on proteomic methodologies are included in the Supplemental Materials and Methods section.

### 4.5. Antioxidant Metabolomic Analyses

Recovery standards were added prior to the extraction process for quality control (QC) purposes. Sample preparation was conducted by a series of organic and aqueous extractions to remove the protein fraction while allowing maximum recovery of small molecules. After removing the organic solvent, each sample was then frozen, dried under vacuum, and prepared for either LC/MS (oxidized glutathione) or GC/MS (ascorbate and α-tocopherol). Details on the methodology used for the metabolomic analyses are included in the Supplemental Materials and Methods.

### 4.6. Nitrate Reductase and Nitrite Reductase Activity Assays

Nitrate reductase activity assays were performed as reported [17] with slight modifications. Leaves were homogenized in extraction buffer (250 mM Tris–HCl (pH 8.0), 1 mM EDTA, 1 μM Na_2_MoO_4_, 5 μM flavin adenine dinucleotide, 3 mM dithiothreitol, 1% BSA, 12 mM β-mercaptoethanol and 250 μM PMSF). After centrifugation at 13,000 rpm for 5 min, supernatants were collected and added to reaction buffer (40 mM NaNO_3_, 80 mM Na_2_HPO_4_, 20 mM NaH_2_PO_4_ (pH 7.5) and 0.2 mM NADH). Assays included 20 μg of protein extracts in 250 μL total volume and were performed for 30 min at 25 °C. The reaction was stopped by the addition of 1% sulphanilamide and 0.05% *N*-(1-napthyl) ethylenediamine hydrochloride. When indicated, 1 mM methyl viologen or bromophenol blue reduced with 5 mM sodium dithionite were used as artificial electron donors to measure terminal NR activity. Nitrite reductase activities were assayed as previously reported [41] with slight modifications. Assays included 50 μg of protein extracts in 250 μL total volume by using dithionite-reduced methyl viologen as an electron donor.

### 4.7. Measurement of Endogenous NO Content

The endogenous levels of NO in shoots and roots of 12-day old seedlings were determined by staining with 10 μM 4-amino-5-methylamino-2′,7′-difluorofluorescein diacetate (DAF-FM DA) fluorescein as described [42] with slight modifications. Fluorescence was detected by confocal microscopy with a CLSM LEICA TCS SP5, using unchanged parameters for every measurement. The specificity of NO-related fluorescence detection was assessed by treatment with 0.5 mM of the NO scavenger 2-phenyl-4,4,5,5-tetramethylimidazoline-1-oxyl-3-oxide (cPTIO, Sigma-Merck, Darmstadt, Germany). The DAF-FM DA fluorescence intensities were analyzed using Adobe Photoshop by quantifying green pixels in 3 to 6 replicate images taken from independent plants in at least 3 different pots for every genotype and condition.

### 4.8. Protein Sequence Analyses and 3D Structure Modelling

Multiple protein sequence alignments were performed by using Clustal Omega (https://www.ebi.ac.uk/Tools/msa/clustalo/). Protein Database Bank files (https://www.rcsb.org/) were processed with Yasara software (www.yasara.org/).

## Data Availability

Not applicable.

## Supplementary Materials

Supplementary Materials can be found at https://www.mdpi.com/1422-0067/22/2/549/s1. Figure S1: NADH- MVH- and BPBH-NR activity in NO-treated plant extracts; Figure S2: Location of identified post-translational modifications and functional domains in NIA1 and NIA2; Figure S3: Effect of NO and proteasome inhibitor MG132 on NR protein (a) and NIA1/NR1 and NIA2/NR2 transcript levels (b). 12-day old seedlings were treated with 0.2 mM MG132 and/or exposed to a 300 ppm NO pulse for 5 min as indicated. Samples for protein and RNA extraction were collected by 1 h after treatments. Transcript levels are relative to those detected in untreated plants (Control) and represent the mean of three independent replicates ± standard error. Table S1: Genes similarly regulated in NO-treated wild type and untreated NO-deficient mutant plants; Table S2: List of peptides identified in 35S:3xHA-NIA1 plants carrying a single nitration, S-nitrosation or ubiquitylation; Table S3: List of peptides identified in 35S:3xHA-NIA2 plants carrying a single nitration, S-nitrosation or ubiquitylation; Table S4: Oligonucleotides used in this work; Supplementary Materials and Methods.

## References

[1] Hancock J.T., Neill S.J. (2019). Nitric Oxide: Its Generation and Interactions with Other Reactive Signaling Compounds. Plants.

[2] Foresi N., Correa-Aragunde N., Parisi G., Caló G., Salerno G., Lamattina L. (2010). Characterization of a nitric oxide synthase from the plant kingdom: NO generation from the green alga *Ostreococcus tauri* is light irradiance and growth phase dependent. Plant Cell.

[3] Weisslocker-Schaetzel M., André F., Touazi N., Foresi N., Lembrouk M., Dorlet P., Frelet-Barrand A., Lamattina L., Santolini J. (2017). The NOS-like protein from the microalgae *Ostreococcus tauri* is a genuine and ultrafast NO-producing enzyme. Plant Sci..

[4] Santolini J., André F., Jeandroz S., Wendehenne D. (2017). Nitric oxide synthase in plants: Where do we stand?. Nitric Oxide.

[5] Solomonson I.P., Barber M.J. (1990). Assimilatory nitrate reductase: Functional properties and regulation. Ann. Rev. Plant Physiol. Plant Mol. Biol..

[6] Rockel P., Strube F., Rockel A., Wildt J., Kaiser W.M. (2002). Regulation of nitric oxide (NO) production by plant nitrate reductase in vivo and in vitro. J. Exp. Bot..

[7] Bender D., Schwarz G. (2018). Nitrite-dependent nitric oxide synthesis by molybdenum enzymes. FEBS Lett..

[8] Gupta K.J., Igamberdiev A.U. (2011). The anoxic plant mitochondrion as a nitrite: NO reductase. Mitochondrion.

[9] Cheng C.L., Dewdney J., Nam H.G., den Boer B.G., Goodman H.M. (1988). A new locus (*NIA 1*) in *Arabidopsis thaliana* encoding nitrate reductase. EMBO J..

[10] Wilkinson J.Q., Crawford N.M. (1991). Identification of the Arabidopsis *CHL3* gene as the nitrate reductase structural gene *NIA2*. Plant Cell.

[11] Costa-Broseta A., Castillo M.C., León J. (2020). Nitrite reductase 1 is a target of nitric oxide-mediated post-translational modifications and controls nitrogen flux and growth in Arabidopsis. Int. J. Mol. Sci..

[12] Kaiser W.M., Huber S.C. (2001). Post-translational regulation of nitrate reductase: Mechanism, physiological relevance and environmental triggers. J. Exp. Bot..

[13] Lillo C., Meyer C., Lea U.S., Provan F., Oltedal S. (2004). Mechanism and importance of post-translational regulation of nitrate reductase. J. Exp. Bot.

[14] MacKintosh C., Meek S.E. (2001). Regulation of plant NR activity by reversible phosphorylation, 14-3-3 proteins and proteolysis. Cell. Mol. Life Sci..

[15] Huber J.L., Huber S.C., Campbell W.H., Redinbaugh M.G. (1992). Reversible light/dark modulation of spinach leaf nitrate reductase activity involves protein phosphorylation. Arch. Biochem. Biophys..

[16] Weiner H., Kaiser W.M. (1999). 14-3-3 proteins control proteolysis of nitrate reductase in spinach leaves. FEBS Lett..

[17] Park B., Song J.T., Seo H.S. (2011). Arabidopsis nitrate reductase activity is stimulated by the E3 SUMO ligase AtSIZ1. Nat. Commun..

[18] Campbell W.H., Kinghorn K.R. (1990). Functional domains of assimilatory nitrate reductases and nitrite reductases. Trends Biochem. Sci..

[19] Crawford N.M., Smith M., Bellissimo D., Davis R.W. (1988). Sequence and nitrate regulation of the *Arabidopsis thaliana* mRNA encoding nitrate reductase, a metalloflavoprotein with three functional domains. Proc. Natl. Acad. Sci. USA.

[20] Hyde G.E., Wilberding J.A., Meyer A.L., Campbell E.R., Campbell W.H. (1989). Monoclonal antibody-based immunoaffinity chromatography for purifying corn and squash NADH: Nitrate reductases. Evidence for an interchain disulfide bond in nitrate reductase. Plant Mol. Biol..

[21] Castillo M.C., Coego A., Costa-Broseta Á., León J. (2018). Nitric oxide responses in Arabidopsis hypocotyls are mediated by diverse phytohormone pathways. J. Exp. Bot..

[22] Gibbs D.J., Md Isa N., Movahedi M., Lozano-Juste J., Mendiondo G.M., Berckhan S., Marín-de la Rosa N., Vicente Conde J., Sousa Correia C., Pearce S.P. (2014). Nitric oxide sensing in plants is mediated by proteolytic control of group VII ERF transcription factors. Mol. Cell.

[23] Kalakoutskii K.L., Fernández E. (1995). *Chlamydomonas reinhardtii* nitrate reductase complex has 105 kDa subunits in the wild-type strain and a structural mutant. Plant Sci..

[24] Perazzolli M., Dominici P., Romero-Puertas M.C., Zago E., Zeier J., Sonoda M., Lamb C., Delledonne M. (2004). Arabidopsis nonsymbiotic hemoglobin AHb1 modulates nitric oxide bioactivity. Plant Cell.

[25] Hill R., Hargrove M., Arredondo-Peter R. (2016). Phytoglobin: A novel nomenclature for plant globins accepted by the globin community at the 2014 XVIII conference on Oxygen-Binding and Sensing Proteins. F1000Research.

[26] Kumar N., Astegno A., Chen J., Giorgetti A., Dominici P. (2016). Residues in the Distal Heme Pocket of Arabidopsis Non-Symbiotic Hemoglobins: Implication for Nitrite Reductase Activity. Int. J. Mol. Sci..

[27] Mehterov N., Balazadeh S., Hille J., Toneva V., Mueller-Roeber B., Gechev T. (2012). Oxidative stress provokes distinct transcriptional responses in the stress-tolerant *atr7* and stress-sensitive *loh2 Arabidopsis thaliana* mutants as revealed by multi-parallel quantitative real-time PCR analysis of ROS marker and antioxidant genes. Plant Physiol. Biochem..

[28] Matsuo M., Johnson J.M., Hieno A., Tokizawa M., Nomoto M., Tada Y., Godfrey R., Obokata J., Sherameti I., Yamamoto Y.Y. (2015). High REDOX RESPONSIVE TRANSCRIPTION FACTOR1 Levels Result in Accumulation of Reactive Oxygen Species in Arabidopsis thaliana Shoots and Roots. Mol. Plant.

[29] Szarka A., Tomasskovics B., Bánhegyi G. (2012). The ascorbate-glutathione-α-tocopherol triad in abiotic stress response. Int. J. Mol. Sci..

[30] Astier J., Gross I., Durner J. (2018). Nitric oxide production in plants: An update. J. Exp. Bot..

[31] León J., Costa-Broseta Á. (2020). Present knowledge and controversies, deficiencies and misconceptions on nitric oxide synthesis, sensing and signaling in plants. Plant Cell Environ..

[32] Kim J.Y., Park B.S., Park S.W., Lee H.Y., Song J.T., Seo H.S. (2018). Nitrate Reductases Are Relocalized to the Nucleus by AtSIZ1 and Their Levels Are Negatively Regulated by COP1 and Ammonium. Int. J. Mol. Sci..

[33] Cheng C.L., Acedo G.N., Dewdney J., Goodman H.M., Conkling M.A. (1991). Differential expression of the two Arabidopsis nitrate reductase genes. Plant Physiol..

[34] Mohn M.A., Thaqi B., Fischer-Schrader K. (2019). Isoform-Specific NO Synthesis by Arabidopsis thaliana Nitrate Reductase. Plants.

[35] Chamizo-Ampudia A., Sanz-Luque E., Llamas Á., Ocaña-Calahorro F., Mariscal V., Carreras A., Barroso J.B., Galván A., Fernández E. (2016). A dual system formed by the ARC and NR molybdoenzymes mediates nitrite-dependent NO production in Chlamydomonas. Plant Cell Environ..

[36] Sadanandom A., Bailey M., Ewan R., Lee J., Nelis S. (2012). The ubiquitin-proteasome system: Central modifier of plant signalling. New Phytol..

[37] Castillo M.C., Lozano-Juste J., González-Guzmán M., Rodriguez L., Rodriguez P.L., León J. (2015). Inactivation of PYR/PYL/RCAR ABA receptors by tyrosine nitration may enable rapid inhibition of ABA signaling by nitric oxide in plants. Sci. Signal..

[38] Clough S.J., Bent A.F. (1998). Floral dip: A simplified method for Agrobacterium-mediated transformation of *Arabidopsis thaliana*. Plant J..

[39] Davenport S., Le Lay P., Sanchez-Tamburrrino J.P. (2015). Nitrate metabolism in tobacco leaves overexpressing Arabidopsis nitrite reductase. Plant Physiol. Biochem..

[40] Blume Y.B. (2020). A journey through a plant cytoskeleton: Hot spots in signaling and functioning. Cell Biol. Int..

[41] Takahashi M., Sasaki Y., Ida S., Morikawa H. (2001). Nitrite reductase gene enrichment improves assimilation of NO_2_^−^ in Arabidopsis. Plant Physiol..

[42] Guo F.Q., Okamoto M., Crawford N.M. (2003). Identification of a plant nitric oxide synthase gene involved in hormonal signaling. Science.

