# Supplementary material for: Post-Translational Modifications of Nitrate Reductases Autoregulates Nitric Oxide Biosynthesis in Arabidopsis
Source: Int J Mol Sci. 2021 Jan 7;22(2):549. doi: 10.3390/ijms22020549 (PMC7827142; doi:10.3390/ijms22020549)
Supplement: Supplementary file 1 [file ijms-22-00549-s001.pdf]

Supplementary data

Post-Translational Modifications of Nitrate Reductases Autoregulates Nitric Oxide Biosynthesis in Arabidopsis

Álvaro Costa-Broseta, MariCruz Castillo and José León*

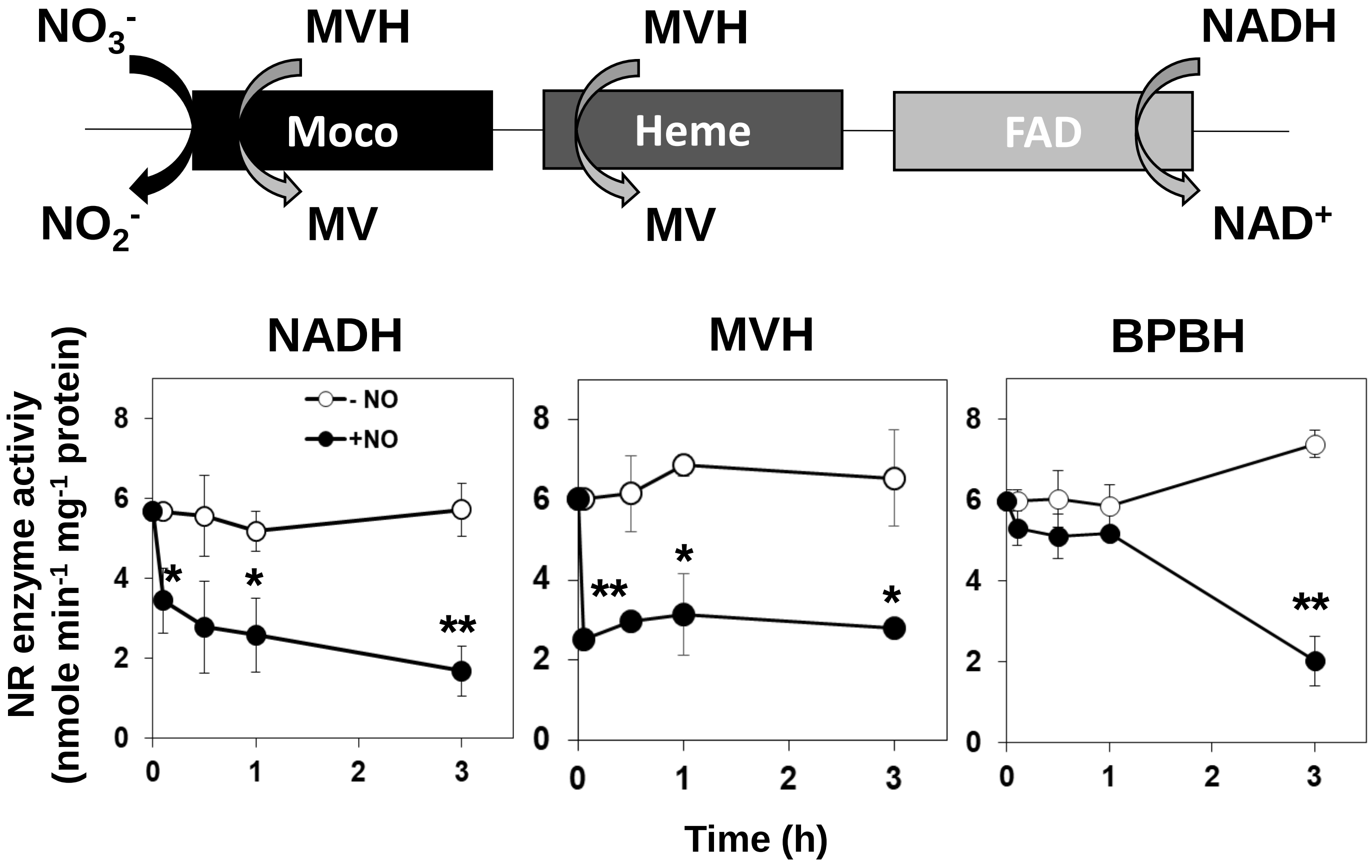

Figure S1. NADH- MVH- and BPBH-NR activity in NO-treated plant extracts. NR activity was assayed with NADH, reduced methyl viologen (MVH) or reduced bromophenol blue (BPBH) as donors passing electrons to flavin (FAD), heme-Molybdenum Cofactor (Moco) or Moco, respectively, as shown in diagram on top.

```
P11832|NIA1_ARATH      MATSVDNRHY----PTMNGVAHAFKPPLVPSPRSFDRHRHQNQTLDVILTETKIVKETEV 56
P11035|NIA2_ARATH      MAASVDNRQYARLEPGLNGVVRSYKPPVPGRSDSPKAHQNQTTNQTVFLKPAKVHDDDE- 59
                       **:*****:*    * :***.:::***:    . *  . *::*. .   *:*. :*: .: *

P11832|NIA1_ARATH      ITTVVDSYDDSSSDDEDESHNRNVPYYKELVKKSNSDLEPSILDPRDESTADSWIQRNSS 116
P11035|NIA2_ARATH      ---------DVSSEDENETHNSNAVYYKEMIRKSNAELEPSVLDPRDEYTADSWIERNPS 110
                                * **:**:*:** *. ****:::***::****:****** ******:**.*

P11832|NIA1_ARATH      MLRLTGKHPFNAEAPLPRLMHHGFITPVPLHYVRNHGAVPKANWSDWSIEITGLVKRPAK 176
P11035|NIA2_ARATH      MVRLTGKHPFNSEAPLNRLMHHGFITPVPLHYVRNHGHVPKAQWAEWTVEVTGFVKRPMK 170
                       *:*********:**** ******************* ****:*::*::*:**:**** *

P11832|NIA1_ARATH      FTMEELISEFPSREFPVTLVCAGNRRKEQNMVKQTIGFNWGSAGVSTSLWKGIPLSEILR 236
P11035|NIA2_ARATH      FTMDQLVSEFAYREFAATLVCAGNRRKEQNMVKKSKGFNWGSAGVSTSVWRGVPLCDVLR 230
                       ***::*:***. ***..****************:: ************:*:*:**.::**

P11832|NIA1_ARATH      RCGIYSRRGGALNVCFEGAEDLPGGG---GSKYGTSIKKEMAMDPARDIILAYMQNGELL 293
P11035|NIA2_ARATH      RCGIFSRKGGALNVCFEGSEDLPGGAGTAGSKYGTSIKKEYAMDPSRDIILAYMQNGEYL 290
                       ****:**:*********:******.    *********** ****:*********** *

P11832|NIA1_ARATH      TPDHGFPVRVIVPGFIGGRMVKWLKRIIVTPQESDSYYHYKDNRVLPSLVDAELANSEAW 353
P11035|NIA2_ARATH      TPDHGFPVRIIIPGFIGGRMVKWLKRIIVTTKESDNFYHFKDNRVLPSLVDAELADEEGW 350
                       *********:*:*****************.:***.:**:**************:.*.*

P11832|NIA1_ARATH      WYKPEYIINELNINSVITTPGHAEILPINAFTTQKPYTLKGYAYSGGGKKVTRVEVTLDG 413
P11035|NIA2_ARATH      WYKPEYIINELNINSVITTPCHEEILPINAFTTQRPYTLKGYAYSGGGKKVTRVEVTVDG 410
                       ******************* * ***********:*********************:**

P11832|NIA1_ARATH      GDTWSVCELDHQEKPNKYGKFWCWCFWSLDVEVLDLLSAKDVAVRAWDESFNTQPDKLIW 473
P11035|NIA2_ARATH      GETWNVCALDHQEKPNKYGKFWCWCFWSLEVEVLDLLSAKEIAVRAWDETLNTQPEKMIW 470
                       *:**.** *********************:**********::*******::****:*:**

P11832|NIA1_ARATH      NLMGMMNNCWFRIRTNVCKPHRGEIGIVFEHPTRPGNQSGGWMAKERQLEISSESNNTLK 533
P11035|NIA2_ARATH      NLMGMMNNCWFRVKTNVCKPHKGEIGIVFEHPTLPGNESGGWMAKERHLEKSADAPPSLK 530
                       ************::*******:*********** ***:*********:** *:::  :**

P11832|NIA1_ARATH      KSVSSPFMNTASKMYSISEVRKHNTADSAWIIVHGHIYDCTRFLKDHPGGTDSILINAGT 593
P11035|NIA2_ARATH      KSVSTPFMNTTAKMYSMSEVKKHNSADSCWIIVHGHIYDCTRFLMDHPGGSDSILINAGT 590
                       ****:*****::****:***:***:*** *************** *****:*********

P11832|NIA1_ARATH      DCTEEFEAIHSDKAKKLLEDYRIGELITTGYDS---SPNVSVHGASNFGPLLAPIKELTP 650
P11035|NIA2_ARATH      DCTEEFEAIHSDKAKKMLEDYRIGELITTGYSSDSSSPNNSVHGSSAVFSLLAPIGEATP 650
                       *********************:*************.*    *** ****:*  . .***** * **

P11832|NIA1_ARATH      QKNIALVNPREKIPVRLIEKTSISHDVRKFRFALPSEDQQLGLPVGKHVFVCANINDKLC 710
P11035|NIA2_ARATH      VRNLALVNPRAKVPVQLVEKTSISHDVRKFRFALPVEDMVLGLPVGKHIFLCATINDKLC 710
                        :*:****** *:**:*:***************** **   ********:*:** .******

P11832|NIA1_ARATH      LRAYTPTSAIDAVGHIDLVVKVYFKDVHPRFPNGGLMSQHLDSLPIGSMIDIKGPLGHIE 770
P11035|NIA2_ARATH      LRAYTPSSTVDVVGYFELVVKIYFGGVHPRFPNGGLMSQYLDSLPIGSTLEIKGPLGHVE 770
                       ******:*::*.**:::****:**  .*************:********* ::*******:*

P11832|NIA1_ARATH      YKGKGNFLVSGKPKFAKKLAMLAGGTGITPIYQIIQSILSDPEDETEMYVVYANRTEDDI 830
P11035|NIA2_ARATH      YLGKGSFTVHGKPKFADKLAMLAGGTGITPVYQIIQAILKDPEDETEMYVIYANRTEEDI 830
                       * ***.* * ******.*************:*****:**.**********:******:**

P11832|NIA1_ARATH      LVREELEGWASKHKERLKIWYVVEIAKEGWSYSTGFITEAVLREHIPEGLEGESLALACG 890
P11035|NIA2_ARATH      LLREELDGWAEQYPDRLKVWYVVESAKEGWAYSTGFISEAIMREHIPDGLDGSALAMACG 890
                       *:****:***.:: :***:***** *****:******:**::*****:**:* .:**:***

P11832|NIA1_ARATH      PPPMIQFALQPNLEKMGYNVKEDLLIF 917
P11035|NIA2_ARATH      PPPMIQFAVQPNLEKMQYNIKEDFLIF 917
                       ********:******* **:***:***
```

Cytochrome b5 heme-binding domain

FAD-binding FR-type domain

MoCo binding

Fe Hemo binding

Sumoylation site YKPE

Figure S2. Location of identified post-translational modifications and functional domains in NIA1 and NIA2. Nitrated Y (red), aminated Y (magenta), Y identified as both nitrated and aminated (purple) in different peptides, S-nitrosated C (blue), ubiquitylated K (green), and oxidized M (turquoise) residues of NIA1 and NIA2 proteins.

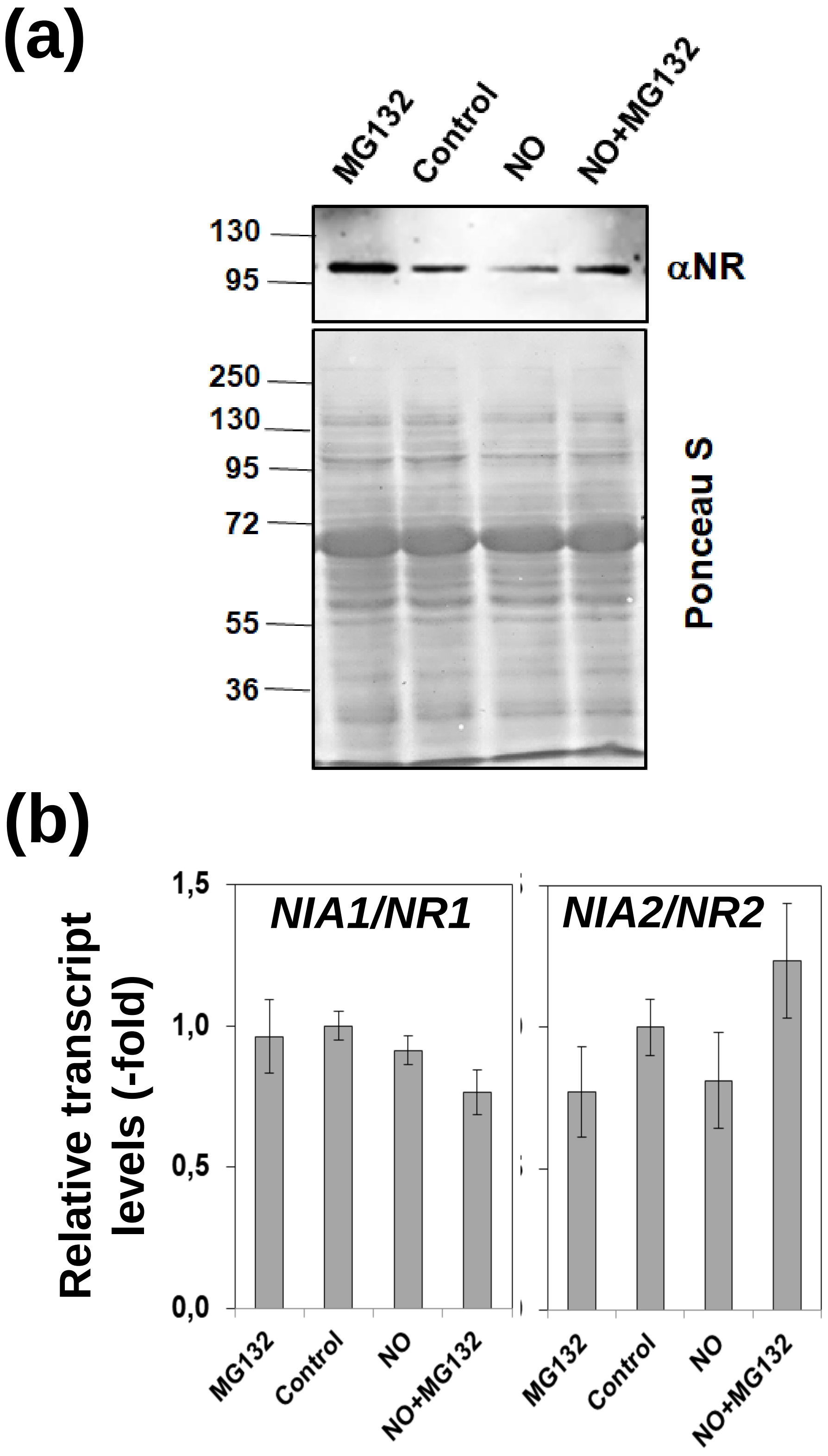

Figure S3. Effect of NO and proteasome inhibitor MG132 on NR protein (a) and NIA1/NR1 and NIA2/NR2 transcript levels (b). 12-day old seedlings were treated with 0.2 mM MG132 and/or exposed to a 300 ppm NO pulse for 5 min as indicated. Samples for protein and RNA extraction were collected by 1 h after treatments. Transcript levels are relative to those detected in untreated plants (Control) and represent the mean of three independent replicates ± standard error.

Supplemental Table S1. Genes similarly regulated in NO-treated wild type and untreated NO-deficient mutant plants. Samples were harvested 12 h after dawn of day 12 after sowing and seedlings were grown under long days (16 h light / 8 h darkness) photoperiodic conditions. Compared analysis of the transcriptomes of 12-day old seedlings from wild type Col-0 treated with a pulse of 300 ppm NO during 5 min and samples collected after 60 min vs untreated control seedlings or *nia1,2noa1-2* vs Col-0 was performed. Linear model methods (LiMMA) were used for determining differentially expressed genes. To control the false-discovery rate (FDR), P-values were corrected using the method of Benjamini and Hochberg (1995).

**NO**		***nia1,2noa1-2***			
**Fold change**	**p-value FDR (LiMMA)**	**Fold change**	**p-value FDR (LiMMA)**	**AGI code**	**GENE_INFO**
12.12	0.00013825	2.92	0.00019885	AT1G63940.3	MDAR6 | monodehydroascorbate reductase
8.19	0.00394474	1.67	0.01109096	AT4G24110.1	unknown protein
6.59	0.00294186	1.95	0.03280612	AT1G76650.1	CML38 | calcium-binding EF hand family
6.57	0.01083634	2.64	0.01140437	AT3G49620.1	DIN11 | DIN11 (DARK INDUCIBLE 11)
6.49	0.003464	3.06	0.00045948	AT2G16060.1	AHB1/GLB1 (ARABIDOPSIS HEMOGLOBIN 1)
6.42	0.00830701	1.54	0.01415087	AT1G65390.2	ATPP2-A5 (ARABIDOPSIS T. PHLOEM PROTEIN 2 A5)
6.05	0.0021898	1.93	0.01526389	AT5G45340.2	CYP707A3; (+)-abscisic acid 8'-hydroxylase
5.83	0.00784132	1.54	0.01415087	AT1G65390.1	ATPP2-A5 (ARABIDOPSIS T. PHLOEM PROTEIN 2 A5)
5.8	0.00356968	1.95	0.03280612	AT1G76650.2	CML38 | calcium-binding EF hand family
5.56	0.00326244	2.45	0.01481085	AT3G10930.1	unknown protein
5.35	0.00429467	1.34	0.04312723	AT5G67080.1	MAPKKK19
5.31	0.00266801	1.93	0.01526389	AT5G45340.1	CYP707A3; (+)-abscisic acid 8'-hydroxylase
5.31	0.0026004	1.74	>0.05	AT5G51190.1	ERF105_AP2 domain-containing transcription factor
5.1	0.01873743	1.96	0.0343714	AT1G69930.1	ATGSTU11 (GLUTATHIONE S-TRANSFERASE TAU 11)
4.5	0.003464	1.97	0.02057983	AT2G24600.2	ankyrin repeat family protein
4.25	0.00294186	1.97	0.02057983	AT2G24600.3	ankyrin repeat family protein
4.23	0.00294186	1.82	0.0330492	AT2G32030.1	GCN5-related N-acetyltransferase (GNAT) family protein
4.22	0.00309366	1.75	0.00050093	AT1G17380.1	JAZ5. TIFY11A (JASMONATE-ZIM-DOMAIN PROTEIN 5)
4.22	0.00310291	1.48	0.04160113	AT3G25780.1	AOC3 (ALLENE OXIDE CYCLASE 3)
4.11	0.00229082	4.53	0.00596634	AT3G50930.1	BCS1 (CYTOCHROME BC1 SYNTHESIS)
3.99	0.00162136	2.1	0.0101275	AT1G61340.1	F-box family protein
3.86	0.00402989	2.01	0.00012475	AT5G14700.1	cinnamoyl-CoA reductase-related
3.75	0.00306866	2.45	0.00759765	AT1G72520.1	lipoxygenase. putative
3.74	0.01218332	2.25	0.0005471	AT4G10270.1	wound-responsive family protein
3.69	0.00102599	2.04	0.00551881	AT4G31800.1	WRKY18; transcription factor
3.58	0.01399612	1.4	0.02430907	AT5G39890.1	unknown protein
3.45	0.0172413	6.05	0.00107631	AT5G39580.2	peroxidase. putative
3.44	0.00221781	1.63	0.0564309	AT4G17490.1	ATERF6 (Ethylene responsive element binding factor 6)
3.38	0.0182418	6.05	0.00107631	AT5G39580.1	peroxidase. putative
3.28	0.00394474	2.12	0.00158597	AT3G02550.1	LBD41 (LOB DOMAIN-CONTAINING PROTEIN 41)
3.21	0.00495528	25.22	0.00000026	AT3G25790.1	Transcription factor HHO1. myb family
3.15	0.00420866	1.97	0.02057983	AT2G24600.1	ankyrin repeat family protein
3.14	0.00309366	3.82	0.00082371	AT1G78050.1	PGM (phosphoglycerate/bisphosphoglycerate mutase)
3.13	0.00306866	2.06	0.00010002	AT4G18340.1	glycosyl hydrolase family 17 protein
3.07	0.00309366	2.44	0.02386155	AT1G80840.1	WRKY40; transcription factor

2.92	0.0385492	1.78	0.02979511	AT4G35180.1	LHT7 (Lys/His transporter 7)
2.9	0.00394838	1.81	0.02864358	AT5G27420.1	E3 ubiquitin-protein ligase ATL31
2.83	0.00290947	2.2	0.00313122	AT5G04340.1	ZAT6 (ZINC FINGER OF ARABIDOPSIS THALIANA 6)
2.81	0.01044382	1.84	0.03540049	AT1G19380.1	unknown protein
2.78	0.00309366	9.62	0.00001112	AT5G66650.1	unknown protein
2.74	0.00326511	3.25	0.01709944	AT1G27730.1	ZAT10 | STZ (salt tolerance zinc finger)
2.66	0.01481975	1.6	0.03618458	AT5G10130.1	pollen Ole e 1 allergen and extensin family protein
2.66	0.00309366	2.65	0.00010149	AT5G07440.3	GDH2 (GLUTAMATE DEHYDROGENASE 2)
2.65	0.00229082	1.57	0.01553525	AT4G27652.1	unknown protein
2.64	0.00565467	3.76	0.00889636	AT5G57220.1	CYP81F2
2.47	0.0107702	2.88	0.00730021	AT1G19020.1	unknown protein
2.29	0.00326047	2.3	0.00076042	AT1G32920.1	unknown protein
2.09	0.01024343	2.5	0.01480833	AT2G22500.1	DIC1 | UCP5 (UNCOUPLING PROTEIN 5)
2.03	0.0063438	2.51	0.00403348	AT4G21410.1	protein kinase family protein
-3.04	0.00229082	-1.48	0.00167968	AT2G03530.1	UPS2 (UREIDE PERMEASE 2)
-3.09	0.00367141	-1.47	0.02463619	AT1G01390.1	UDP-glucoronosyl/UDP-glucosyl transferase family protein
-3.19	0.01226263	-1.74	0.00053262	AT3G15840.4	PIFI (post-illumination chlorophyll fluorescence increase)
-3.23	0.00315341	-2.42	0.00050951	AT2G29290.2	tropinone reductase. putative
-3.24	0.00451369	-1.76	0.01215664	AT5G28910.1	unknown protein
-3.25	0.00413041	-1.45	0.00590768	AT1G69523.1	UbiE/COQ5 methyltransferase family protein
-3.26	0.00562376	-8.95	0.00002154	AT1G53870.2	unknown protein
-3.28	0.00326511	-1.78	0.00197837	AT3G01960.1	unknown protein
-3.29	0.00507786	-1.84	0.00266912	AT3G28540.1	AAA-type ATPase family protein
-3.37	0.00356968	-2.13	0.00025003	AT2G03710.3	AGL3 | SEP4 (SEPALLATA 4)
-3.39	0.03871652	-1.24	0.01853354	AT1G44575.2	PSBS | NPQ4 (NONPHOTOCHEMICAL QUENCHING)
-3.39	0.00358222	-2.31	0.02241089	AT4G14400.1	ACD6 (ACCELERATED CELL DEATH 6)
-3.41	0.02152208	-2.12	0.00438396	AT1G18710.1	AtMYB47 (myb domain protein 47)
-3.41	0.00294186	-1.22	0.03399151	AT3G59580.1	RWP-RK domain-containing protein
-3.44	0.00404197	-1.76	0.01215664	AT5G28910.2	unknown protein
-3.48	0.03715538	-1.39	0.03190261	AT2G45560.2	CYP76C1
-3.48	0.0055488	-1.66	0.00086056	AT3G01480.2	CYP38 (cyclophilin 38); peptidyl-prolyl cis-trans isomerase
-3.54	0.00411275	-2.05	0.00278555	AT1G75250.2	ATRL6 (ARABIDOPSIS RAD-LIKE 6)
-3.57	0.00326047	-1.8	0.00294101	AT5G56850.1	unknown protein
-3.61	0.00627604	-5.47	0.00003747	AT4G26530.2	fructose-bisphosphate aldolase. putative
-3.61	0.00737142	-1.45	0.02514399	AT5G38510.1	rhomboid family protein
-3.64	0.02093813	-1.7	0.00246097	AT1G18730.4	NDF6 (NDH DEPENDENT FLOW 6)
-3.66	0.00371237	-1.76	0.00056152	AT3G09160.1	RNA recognition motif (RRM)-containing protein
-3.68	0.0029534	-1.33	0.03402035	AT1G32780.1	alcohol dehydrogenase. putative
-3.7	0.00309366	-1.41	0.03262415	AT1G22590.2	AGL87 | MADS-box family protein
-3.71	0.00965735	-1.64	0.00507932	AT1G76190.1	auxin-responsive family protein
-3.77	0.00860028	-1.47	0.02464749	AT1G78290.2	serine/threonine protein kinase. putative
-3.78	0.00309366	-2.16	0.00008759	AT1G65790.1	ARK1 (A. THALIANA RECEPTOR KINASE 1)
-3.94	0.00367141	-1.45	0.02514399	AT5G38510.2	rhomboid family protein
-3.95	0.00572337	-1.54	0.00258045	AT5G28020.1	CYSD2 (CYSTEINE SYNTHASE D2)
-3.97	0.00417125	-1.48	0.00399672	AT3G13065.1	SRF4 (STRUBBELIG-RECEPTOR FAMILY 4)
-3.99	0.01165814	-1.99	>0.05	AT2G14560.1	LURP1 (late upregulated in response to Hyaloperonospora)
-4.18	0.00229082	-5.5	0.00001112	AT3G46370.1	leucine-rich repeat protein kinase
-4.32	0.00663139	-1.55	0.03437374	AT5G65890.1	ACR1 (ACT Domain Repeat 1)
-4.34	0.0034714	-3.15	0.00022666	AT4G13830.1	J20 (DNAJ-LIKE 20); heat shock protein binding
-4.38	0.04799851	-2.19	0.0105972	AT1G07050.1	CONSTANS-like protein-related
-4.46	0.00356968	-2.76	0.00084849	AT5G10250.1	DOT3 (DEFECTIVELY ORGANIZED TRIBUTARIES 3)
-4.54	0.00323071	-1.33	0.02514517	AT1G52510.2	hydrolase. alpha/beta fold family protein

-5.29	0.00294186	-1.6	0.00066382	AT1G22370.1	AtUGT85A5 (UDP-glucosyl transferase 85A5)
-5.53	0.00162136	-1.7	0.00174842	AT3G13062.2	unknown protein
-5.8	0.00346132	-1.83	0.00281002	AT2G32290.1	BAM6 (BETA-AMYLASE 6)
-6.49	0.00896923	-2.31	0.02239602	AT2G15020.1	unknown protein

Supplemental Table S2. List of peptides identified in *35S:3xHA-NIA1* plants carrying a single nitration, S-nitrosation or ubiquitylation. Nine independent samples were analyzed and one miss cleavage maximum was allowed.

Sequence	Protein Group Accessions	Modifications	XCorr	MH+ [Da]	ΔM [ppm]	Missed Cleavages
AyTPTSAIDAVGHIDLVVK	P11832	Y2(Amino)	1.55	1985.06394	-3.21	0
EELEGWASk	P11832	K9(GlyGly)	1.20	1162.54106	3.02	0
EGWSySTGFITEAVLR	P11832	Y5(Nitro)	1.93	1860.88237	3.24	0
EGWSySTGFITEAVLR	P11832	Y5(Nitro)	1.50	1860.88201	3.04	0
EQNmVkQTIGFNWGSAGVSTSLWK	P11832	M4(Oxidation); K6(GlyGly)	3.09	2798.34757	-3.29	1
gEIGIVFEHPTRPGNQSGGWMAkER	P11832	N-Term(Acetyl); K23(GlyGly)	1.47	2909.40543	-2.01	1
gKGNFLVSGKPk	P11832	N-Term(Acetyl); K12(GlyGly)	1.22	1387.77024	0.68	1
GKGNFLVSGkPK	P11832	K10(GlyGly)	1.25	1345.75229	-4.78	1
gNFLVSGKPk	P11832	N-Term(Acetyl); K10(GlyGly)	0.95	1202.65190	-0.79	0
gPLGHIEyKGK	P11832	N-Term(Acetyl); Y8(Amino)	0.91	1255.67510	-3.42	1
GPLGHIEyKGK	P11832	Y8(Amino)	1.41	1213.67621	6.08	1
GPLGHIEyKGK	P11832	Y8(Amino)	1.91	1213.67658	6.38	1
hQNQTLDVILTETk	P11832	N-Term(Acetyl); K14(GlyGly)	1.43	1795.91460	-2.19	0
hVFVCANINDk	P11832	N-Term(Acetyl); K11(GlyGly)	1.98	1415.66619	-5.28	0
HVFVcANINDK	P11832	C5(Nitrosyl)	1.17	1288.61601	4.41	0
hVFVCANINDk	P11832	N-Term(Acetyl); K11(GlyGly)	1.16	1415.66557	-5.71	0
hVFVCANINDk	P11832	N-Term(Acetyl); K11(GlyGly)	1.07	1415.66570	-5.63	0
hVFVCANINDk	P11832	N-Term(Acetyl); K11(GlyGly)	1.96	1415.68132	5.41	0
iIVTPQESDSYYHYk	P11832	N-Term(Acetyl); K15(GlyGly)	1.23	1998.93706	-3.66	0
IIVTPQESDSyYHYKDNR	P11832	Y11(Amino)	1.77	2243.08738	6.51	1
iWyVVEIAK	P11832	N-Term(Acetyl); Y3(Nitro)	1.22	1207.63176	-3.34	0
iWYVVEIAk	P11832	N-Term(Acetyl); K9(GlyGly)	1.12	1276.69048	-2.48	0
iWyVVEIAKEGWSYSTGFITEAVLR	P11832	N-Term(Acetyl); Y3(Amino)	3.31	2974.51920	-5.32	1
kEMAMDPAR	P11832	K1(GlyGly)	1.47	1162.54106	5.72	1
KHNTADSAWIIVHGHIYDcTR	P11832	C19(Nitrosyl)	2.04	2466.17154	-0.68	1
kLLEDyR	P11832	N-Term(Acetyl); Y6(Nitro)	1.23	1023.51042	-0.16	1
KSVSSPFMNTASk	P11832	K13(GlyGly)	1.44	1497.73955	1.92	1
KSVSSPFMNTASk	P11832	K13(GlyGly)	1.92	1497.74199	3.55	1
LcLRAYTPTSAIDAVGHIDLVVK	P11832	C2(Nitrosyl)	1.53	2484.31711	-4.39	1
LcLRAYTPTSAIDAVGHIDLVVK	P11832	C2(Nitrosyl)	1.28	2484.31803	-4.02	1
LcLRAYTPTSAIDAVGHIDLVVK	P11832	C2(Nitrosyl)	1.62	2484.31894	-3.65	1
LcLRAYTPTSAIDAVGHIDLVVK	P11832	C2(Nitrosyl)	1.52	2484.31766	-4.17	1
LcLRAYTPTSAIDAVGHIDLVVK	P11832	C2(Nitrosyl)	1.28	2484.32151	-2.62	1
lMHHGFITPVPLHyVRNHGAVPK	P11832	N-Term(Acetyl); Y14(Nitro)	1.63	2707.40308	-0.30	1

lMHHGFITPVPLHyVRNHGAVPK	P11832	N-Term(Acetyl); Y14(Nitro)	2.33	2707.40810	1.55	1
lMHHGFITPVPLHyVRNHGAVPK	P11832	N-Term(Acetyl); Y14(Nitro)	1.63	2707.41005	2.27	1
MVkWLK	P11035;P11832	K3(GlyGly)	1.13	918.52513	2.30	1
mVkWLK	P11035;P11832	N-Term(Acetyl); K3(GlyGly)	1.29	960.53581	2.32	1
mySISEVR	P11832	M1(Oxidation); Y2(Nitro)	0.97	1045.45610	-5.57	0
MySISEVRK	P11832	Y2(Nitro)	1.16	1157.56169	-0.25	1
mySISEVRK	P11832	N-Term(Acetyl); Y2(Nitro)	1.09	1199.57512	2.15	1
MySISEVRK	P11832	Y2(Nitro)	1.14	1157.56133	-0.56	1
MySISEVRK	P11832	Y2(Nitro)	1.17	1157.56267	0.60	1
MySISEVRK	P11832	Y2(Nitro)	1.36	1157.56218	0.17	1
MYSISEVRk	P11832	K9(GlyGly)	1.37	1226.61921	-0.51	1
MySISEVRK	P11832	Y2(Nitro)	1.03	1157.56108	-0.77	1
MySISEVRK	P11832	Y2(Nitro)	1.33	1157.56218	0.17	1
nVPyYK	P11832	N-Term(Acetyl); Y4(Amino)	0.94	840.42888	4.58	0
NVPyYKELVK	P11832	Y4(Amino)	1.52	1267.70073	-3.00	1
NVPyYKELVK	P11832	Y4(Amino)	1.45	1267.70122	-2.61	1
NVPyYKELVK	P11832	Y4(Amino)	1.34	1267.70146	-2.42	1
NVPyYKELVK	P11832	Y4(Amino)	1.38	1267.70122	-2.61	1
NVPyYKELVK	P11832	Y4(Amino)	1.18	1267.70000	-3.57	1
NVPyYKELVK	P11832	Y4(Amino)	1.67	1267.70098	-2.80	1
NVPyYKELVK	P11832	Y4(Amino)	1.21	1267.70049	-3.19	1
qTIGFNWGSAGVSTSLWk	P11832	N-Term(Acetyl); K18(GlyGly)	1.30	2095.02544	0.49	0
RCGIySR	P11832	Y5(Amino)	0.94	869.44560	5.23	1
RGGALNVcFEGAEDLPGGGGSK	P11832	C8(Nitrosyl)	1.66	2119.98722	2.14	1
RIIVTPQESDSYYHyK	P11832	Y15(Nitro)	2.00	2043.98124	2.03	1
RIIVTPQESDSYyHYK	P11832	Y13(Nitro)	1.50	2043.98124	2.03	1
sVSSPFMNTASk	P11832	N-Term(Acetyl); K12(GlyGly)	1.83	1411.64812	-2.94	0
TNVCkPHR	P11832	K5(GlyGly)	1.17	1068.54314	5.97	0
TNVcKPHR	P11832	C4(Nitrosyl)	1.31	983.48076	-3.29	0
TNVCkPHR	P11832	K5(GlyGly)	1.09	1068.54363	6.43	0
TNVCKPHRGEIGIVFEHPTRPGNQSGGWMAk	P11832	K31(GlyGly)	1.47	3517.73838	1.53	1
tSISHDVRk	P11035;P11832	N-Term(Acetyl); K9(GlyGly)	1.22	1198.62146	3.30	1
TSISHDVRk	P11035;P11832	K9(GlyGly)	1.02	1156.59917	-6.73	1
vyFKDVHPR	P11832	N-Term(Acetyl); Y2(Amino)	0.99	1217.63848	-3.39	1
vyFKDVHPR	P11832	N-Term(Acetyl); Y2(Amino)	1.13	1217.63701	-4.59	1
VyFKDVHPR	P11832	Y2(Amino)	1.43	1175.62517	-5.84	1
YGTSIkK	P11035;P11832	K6(GlyGly)	1.04	910.49834	-1.03	1
YGTSIkK	P11035;P11832	K6(GlyGly)	1.19	910.49877	-0.56	1
YGTSIkK	P11035;P11832	K6(GlyGly)	1.30	910.49925	-0.03	1
yGTSIkK	P11035;P11832	N-Term(Acetyl); K6(GlyGly)	1.04	952.50945	-0.42	1

Supplemental Table S3. List of peptides identified in *35S:3xHA-NIA2* plants carrying a single nitration, S-nitrosation or ubiquitylation. Nine independent samples were analyzed and one miss cleavage maximum was allowed.

Sequence	Protein Group Accessions	Modifications	XCorr	MH+ [Da]	ΔM [ppm]	Missed Cleavages
aQWAEWTVEVTGFVKRPMk	P11035	N-Term(Acetyl); K19(GlyGly)	1.65	2419.21880	-1.67	1
AWDETLNTQPEk	P11035	K12(GlyGly)	1.40	1545.71555	-1.61	0
eELDGWAEQyPDRLK	P11035	N-Term(Acetyl); Y10(Nitro)	1.22	1935.86150	-5.42	1
EFAATLVcAGNRR	P11035	C8(Nitrosyl)	2.47	1436.70224	-2.88	1
EFAATLVcAGNRR	P11035	C8(Nitrosyl)	2.01	1436.70087	-3.84	1
EFAATLVcAGNRR	P11035	C8(Nitrosyl)	1.33	1436.70279	-2.50	1
EGWAySTGFISEAIMR	P11035	Y5(Amino)	1.33	1832.86235	-0.72	0
EGWAySTGFISEAIMR	P11035	Y5(Amino)	2.26	1832.86199	-0.92	0
EGWAySTGFISEAIMR	P11035	Y5(Amino)	2.46	1832.86272	-0.52	0
eGWAySTGFISEAIMR	P11035	N-Term(Acetyl); Y5(Nitro)	1.43	1904.85161	1.67	0
EGWAySTGFISEAIMR	P11035	Y5(Amino)	2.12	1832.86455	0.47	0
EGWAySTGFISEAIMR	P11035	Y5(Amino)	2.40	1832.86089	-1.52	0
EGWAySTGFISEAIMR	P11035	Y5(Amino)	2.74	1832.86431	0.34	0
EGWAySTGFISEAIMR	P11035	Y5(Amino)	2.44	1832.86284	-0.46	0
FALPVEDMVLGLPVGk	P11035	K16(GlyGly)	1.36	1798.98630	5.02	0
gGALNVCFEGSEDLPGGAGTAGSKyGTSIK	P11035	N-Term(Acetyl); Y25(Amino)	2.13	2900.37741	1.44	1
GPLGHVEyLGK	P11035	Y8(Nitro)	1.39	1214.61518	-1.06	0
GPLGHVEyLGK	P11035	Y8(Amino)	0.96	1184.64800	4.83	0
GPLGHVEyLGK	P11035	Y8(Nitro)	1.40	1214.61618	-0.23	0
GPLGHVEYLGkGSFTVHGKPK	P11035	K11(GlyGly)	2.00	2322.24845	5.61	1
GVPLcDVLR	P11035	C5(Nitrosyl)	1.09	1000.52715	2.66	0
GVPLcDVLR	P11035	C5(Nitrosyl)	1.02	1000.52867	4.19	0
GVPLcDVLR	P11035	C5(Nitrosyl)	1.10	1000.52593	1.44	0
GVPLcDVLR	P11035	C5(Nitrosyl)	1.20	1000.52867	4.19	0
GVPLcDVLR	P11035	C5(Nitrosyl)	1.22	1000.52892	4.43	0
HIFLCATINDkLCLR	P11035	K11(GlyGly)	1.04	1873.97966	1.10	1
IyFGGVHPR	P11035	Y2(Amino)	1.33	1060.56279	-5.58	0
KEyAMDPSR	P11035	Y3(Amino)	1.17	1111.51592	-3.78	1
lKVWYVVESAk	P11035	N-Term(Acetyl); K11(GlyGly)	1.33	1477.81316	5.52	1
MQyNIK	P11035	Y3(Amino)	1.01	811.41344	0.40	0
mVkWLK	P11035;P11832	N-Term(Acetyl); K3(GlyGly)	1.19	960.53667	3.21	1
MVkWLK	P11035;P11832	K3(GlyGly)	1.13	918.52513	2.30	1
mVkWLK	P11035;P11832	N-Term(Acetyl); K3(GlyGly)	1.29	960.53581	2.32	1
mySMSEVK	P11035	N-Term(Acetyl); Y2(Nitro)	1.21	1061.43083	2.81	0
mySMSEVK	P11035	N-Term(Acetyl); Y2(Nitro)	0.91	1061.42119	-6.28	0

MYSMSEVKk	P11035	K9(GlyGly)	1.11	1216.57427	3.42	1
MYSMSEVKk	P11035	K9(GlyGly)	1.15	1216.57378	3.02	1
rcGIFSR	P11035	N-Term(Acetyl); C2(Nitrosyl)	1.11	909.43932	3.66	1
sADAPPSLkK	P11035	N-Term(Acetyl); K9(GlyGly)	1.15	1169.61638	0.21	1
sADAPPSLkK	P11035	N-Term(Acetyl); K9(GlyGly)	1.24	1169.61760	1.26	1
SDSPKAHQNQTTNQTVFLKPAk	P11035	K22(GlyGly)	1.29	2554.29618	-1.86	1
skGFNWGSAGVSTSVWR	P11035	N-Term(Acetyl); K2(GlyGly)	2.17	1981.95964	4.07	1
skGFNWGSAGVSTSVWR	P11035	N-Term(Acetyl); K2(GlyGly)	1.71	1981.96001	4.25	1
skGFNWGSAGVSTSVWR	P11035	N-Term(Acetyl); K2(GlyGly)	2.06	1981.95854	3.51	1
SkGFNWGSAGVSTSVWR	P11035	K2(GlyGly)	1.12	1939.95024	4.76	1
skGFNWGSAGVSTSVWR	P11035	N-Term(Acetyl); K2(GlyGly)	1.90	1981.96025	4.37	1
skGFNWGSAGVSTSVWR	P11035	N-Term(Acetyl); K2(GlyGly)	2.43	1981.95976	4.13	1
SNAELEPSVLDPRDEyTADSWIER	P11035	Y16(Amino)	1.22	2807.31900	2.50	1
SNAELEPSVLDPRDEyTADSWIER	P11035	Y16(Amino)	1.56	2807.31333	0.48	1
SNAELEPSVLDPRDEyTADSWIER	P11035	Y16(Amino)	1.41	2807.32962	6.28	1
sVSTPFMNTTAKMySMSEVK	P11035	N-Term(Acetyl); Y14(Nitro)	1.25	2326.05136	4.85	1
SyKPPVPGR	P11035	Y2(Nitro)	1.11	1045.54253	-0.03	0
SyKPPVPGR	P11035	Y2(Nitro)	1.19	1045.54216	-0.38	0
SyKPPVPGR	P11035	Y2(Nitro)	1.09	1045.54204	-0.50	0
SyKPPVPGR	P11035	Y2(Amino)	1.15	1015.57488	6.40	0
tSISHDVRk	P11035;P11832	N-Term(Acetyl); K9(GlyGly)	1.22	1198.62146	3.30	1
TSISHDVRk	P11035;P11832	K9(GlyGly)	1.02	1156.59917	-6.73	1
VKTNVCKPHk	P11035	K10(GlyGly)	1.66	1267.70122	5.70	1
vkTNVCKPHK	P11035	N-Term(Acetyl); K2(GlyGly)	1.27	1309.69690	-5.85	1
vKTNVCKPHk	P11035	N-Term(Acetyl); K10(GlyGly)	1.21	1309.69745	-5.43	1
VKTNVCkPHK	P11035	K7(GlyGly)	1.31	1267.70098	5.50	1
vKTNVCkPHK	P11035	N-Term(Acetyl); K7(GlyGly)	1.59	1309.69773	-5.22	1
VKTNVCkPHK	P11035	K7(GlyGly)	1.17	1267.70049	5.12	1
VWYVVESAk	P11035	K9(GlyGly)	1.36	1194.61155	-3.22	0
vWyVVESAK	P11035	N-Term(Acetyl); Y3(Amino)	0.90	1137.60088	6.11	0
YGTSIkK	P11035;P11832	K6(GlyGly)	1.04	910.49834	-1.03	1
YGTSIkK	P11035;P11832	K6(GlyGly)	1.19	910.49877	-0.56	1
YGTSIkK	P11035;P11832	K6(GlyGly)	1.30	910.49925	-0.03	1
yGTSIkK	P11035;P11832	N-Term(Acetyl); K6(GlyGly)	1.04	952.50945	-0.42	1

Supplemental Table S4. Oligonucleotides used in this work.

Name	Sequence (5´to 3´)	AGI	Application
35S-seq	CCTTCGCAAGACCCTTCCTCTA		Cloning pAlligator2
NOS-term-rev	GCAAGACCGGCAACAGGATTCAATC		Cloning pAlligator2
NR1cds-F	ATGGCGACCTCCGTCGATAACCGCCATTATCCC	AT1G77760	Cloning NIA1
NR1cds-R	GAAGATTAAGAGATCCTCCTTCACGTTGTAACC	AT1G77760	Cloning NIA1
seqNR1_F	AGGAGATGGCGATGGATC	AT1G77760	Cloning NIA1
SeqNR1_3rev	GATCGAAGTCTTCTCAATG	AT1G77760	Cloning NIA1
SeqNR1_2fw	GAGCTAATCTCCGAGTTC	AT1G77760	Cloning NIA1
NR2cds-F	ATGGCGGCCTCTGTAGATAATCGCCAATACGCT	AT1G37130	Cloning NIA2
NR2cds-R	GAATATCAAGAAATCCTCCTTGATGTTATATTG	AT1G37130	Cloning NIA2
seqNR2_F	AGAACATGGTGAAGAAGTC	AT1G37130	Cloning NIA2
SeqNR2_2rev	CTAGAACCATATCCTCAAC	AT1G37130	Cloning NIA2
NIR1cds-F	ATGACTTCTTTCTCTCTCACTTTCACATCTCC	AT2G15620	Cloning NIR1
NIR1cds-R	ATCTTCATTCTCTTCTCTTTCTCTAGGCACAG	AT2G15620	Cloning NIR1
seqNIR1_F	GTGGTATGGATAACGTGAG	AT2G15620	Cloning NIR1
qACT2-F	TTGTTCCAGCCCTCGTTTGT	AT3G18780	qRT-PCR
qACT2-R	TGTCTCGTGGATTCCAGCAG	AT3G18780	qRT-PCR
qNIA1-F	AGGTTTGGAAGGCGAATCG	AT1G77760	qRT-PCR
qNIA1-R	TGGCTGCAACGCAAACTG	AT1G77760	qRT-PCR
qNIA2-2F	ACGTCCCTAAAGCCCAATGG	AT1G37130	qRT-PCR
qNIA2-2R	AATTTCATGGGCCGTTTGAC	AT1G37130	qRT-PCR
qRRTF1_F	GTCAGGGTTTTTCCAGTGACAGCA	AT4G34410	qRT-PCR
qRRTF1_R	GTGTCTGAATCCAACCGAGGCATT	AT4G34410	qRT-PCR
qHRS1-F	TCCGAGGACAAGAACACGAAATC	AT1G13300	qRT-PCR
qHRS1-R	TGTCATCGTCTCCTGCTGCAA	AT1G13300	qRT-PCR
qZAT10-F	CCTCAGTGAGGTTTTGGTGGTGGA	AT1G28110	qRT-PCR
qZAT10-R	TCGAGCACTGGACAAAGGGTAAGC	AT1G28110	qRT-PCR
qWRKY70-F	AAAAGATTGGGACCCGTTAA	AT3G56400	qRT-PCR
qWRKY70-R	TGGGAGTTTCTGCGTTGG	AT3G56400	qRT-PCR
qSZF1-F	TCCTCTCCAAGAAACGGCGGATCA	AT3G55980	qRT-PCR
qSZF1-R	TTGAGCTGCAAAGCCGGTGGAGTA	AT3G55980	qRT-PCR
qBNQ2-F	CCGTCGTTCCAACACGGTATCA	AT5G15160	qRT-PCR
qBNQ2-R	CTGCGGCTTGTGGGCTATTAGG	AT5G15160	qRT-PCR
qERF056-F	GCGATATCTCCACGGAGCTGAAAA	AT2G22200	qRT-PCR
qERF056-R	GGGACATTCGAAACCGCCGAAAAA	AT2G22200	qRT-PCR

Supplementary Materials and Methods

1. Proteomic Analyses of Post-translational Modification Sites

Raw data were processed and analyzed by using the Mascot Server v2.4 (Matrix Science) database (FDR < 5%, Arabidopsis thaliana Uniprot database including 13140 proteins, with a mass tolerance of 7ppm for the precursors and 0.5 Da for fragments). A customized database with the 864 proteins identified in the original search was then reanalyzed with SEQUEST searching for the N-terminal modification by acetylation (+42.011 Da) and the dynamic modifications of S-nitrosylation of C (+28.990 Da), nitration of Y (+44.985 Da), amination of Y (+15.011 Da), oxidation of M (+15.995 Da), and GG of K (+114.043 Da) as a mark of ubiquitination after trypsin digestion. Identified peptides were filtered by XCorr (z=2 XCorr>0.9, z=3 XCorr>1.2, z>4 XCorr>1.5) and deltaCN > 0.15. Maximum number of missed cleavages was 3 and the MS and MS2 tolerances were 0.5 and 0.7, respectively. To rule out the chance of false identification of nitration or S nitrosylation modification during MSMS analysis, we run control protein samples immunopurified and digested with trypsin under reducing conditions and no nitroY or nitrosoC containing peptide was identified.

2. LC/MS- and GC/MS-based analyses of the metabolome of Arabidopsis thaliana seedlings

The sample preparation process was carried out using the automated MicroLab STAR® system from Hamilton Company. Recovery standards were added prior to the first step in the extraction process for quality Control (QC) purposes. Sample preparation was conducted by series of organic and aqueous extractions to remove the protein fraction while allowing maximum recovery of small molecules. The resulting extract was divided into two fractions, one for analysis by Liquid Chromatography (LC) and one for analysis by Gas Chromatography (GC). Samples were placed briefly on a TurboVap® (Zymark) to remove the organic solvent. Each sample was then frozen, dried under vacuum and prepared for either LC/MS or GC/MS. The LC/MS portion of the platform was based on a Waters ACQUITY UPLC and a Thermo-Finnigan LTQ mass spectrometer, which consisted of an electrospray ionization (ESI) source and linear ion-trap (LIT) mass analyzer. The sample extract was split into two aliquots, dried, then reconstituted in acidic or basic LC-compatible solvents, each of which contained 11 or more injection standards at fixed concentrations. One aliquot was analyzed using acidic positive ion optimized conditions and the other using basic negative ion optimized conditions in two independent injections using separate dedicated columns. Extracts reconstituted in acidic conditions were gradient eluted using water and methanol both containing 0.1% Formic acid, while the basic extracts, which also used water/methanol, contained 6.5 mM ammonium bicarbonate. The MS analysis alternated between MS and data-dependent MS2 scans using dynamic exclusion. The Thermo-Finnigan LTQ-FT mass spectrometer had a linear ion-trap (LIT) front end and a Fourier transform ion cyclotron resonance (FT-ICR) mass spectrometer back end. For ions with counts greater than 2 million, an accurate mass measurement could be performed. Accurate mass measurements could be made on the parent ion as well as fragments. The typical mass error was less than 5 ppm. Ions with less than two million counts require fragmentation spectra (MS/MS) typically generated in data dependent manner or targeted MS/MS in the case of lower level signals. The samples destined for GC/MS analysis were re-dried under vacuum desiccation for a minimum of 24 hours prior to being derivatized under dried nitrogen using bistrimethyl-silyl-triflouroacetamide (BSTFA). The GC column was 5% phenyl and the temperature ramp is from 40° to 300° C in a 16 minute period. Samples were analyzed on a Thermo-Finnigan Trace DSQ fast-scanning single-quadrupole mass spectrometer using electron impact ionization. The data extraction of the raw mass spec data files yielded information that was loaded into a relational database and manipulated without resorting to BLOB manipulation. Peaks were identified using peak integration software, and component

parts were stored in a separate and specifically designed complex data structure. Compounds were identified by comparison to library entries of more than 1000 commercially available purified standards. The combination of chromatographic properties and mass spectra gave an indication of a match to the specific compound or an isobaric entity. Additional entities could be identified by virtue of their recurrent nature (both chromatographic and mass spectral). A variety of curation procedures were carried out to ensure accurate and consistent identification of true chemical entities, and to remove those representing system artifacts, mis-assignments, and background noise.
